# Optic Neuritis

**Published:** 2010-07

**Authors:** Mary Caitlin Dooley, Rod Foroozan

**Affiliations:** Neuro-Ophthalmology Service, Baylor College of Medicine, Houston, Texas, USA

**Keywords:** Optic Neuritis, Optic Nerve Diseases, Demyelinating Diseases

## Abstract

Demyelinating optic neuritis is the most common cause of unilateral painful visual loss in the United States. Although patients presenting with demyelinating optic neuritis have favorable long-term visual prognosis, optic neuritis is the initial clinical manifestation of multiple sclerosis in 20% of patients. The Optic Neuritis Treatment Trial (ONTT) has helped stratify the risk of developing multiple sclerosis after the first episode of optic neuritis based on abnormal findings on brain MRI. The ONTT also demonstrated that while initial treatment of optic neuritis with intravenous corticosteroids followed by an oral taper accelerates visual recovery, its use does not improve long-term visual outcomes. Long-term treatment with immunomodulating agents such as interferons has been shown to improve clinical outcomes and neuroimaging abnormalities in multiple sclerosis; furthermore interferon use has been associated with decreased risk of subsequent multiple sclerosis in patients with an acute neurologic syndrome. However, several questions regarding the presentation, management, and implications of acute demyelinating optic neuritis remain unanswered.

## INTRODUCTION

A variety of conditions lead to inflammation of the optic nerve (optic neuritis), however, the form associated with demyelination due to multiple sclerosis is most common.^20^ Demyelinating optic neuritis is not only the most common cause of unilateral painful visual loss in the United States and many other parts of the world^1^, but is also a diagnosis that brings very concerning news to an often previously healthy patient. Optic neuritis is the first clinical manifestation of multiple sclerosis in 20% of patients.^1^ Patients with optic neuritis will likely have many questions for the diagnosing physician, whether that be an ophthalmologist, a neurologist, or a primary care physician. It is important that these practitioners have a clear understanding of the condition, how to make the diagnosis, its treatment, and perhaps most importantly, its implications for future management.

## PATHOGENESIS

The pathogenesis of optic neuritis, as with other demyelinating events associated with multiple sclerosis, is not entirely understood. It is believed that some inflammatory process leads to peripheral T-cell activation. These T-cells are thought to cross the blood-brain barrier and cause a delayed type IV hypersensitivity reaction causing destruction of myelin, the insulator and protector of nerves.^1^ It was previously thought that this process only affects myelin and is limited to the white matter. More recent technology, including optical coherence tomography, suggests that the axon of the nerve itself is affected and that the gray matter is also involved.^2^ Clinically, inflammation of the optic nerve translates to visual loss, which is typically followed by visual recovery. This recovery often begins spontaneously, within two to three weeks, and stabilizes over several months.^3^

## CLINICAL FINDINGS

The typical patient who develops acute demyelinating optic neuritis is an otherwise healthy young adult, most often 20 to 45 years of age; women are affected three times more commonly than men.^4^ Children may also develop optic neuritis, but in such cases it is typically related to an infection, often viral, is usually bilateral^5^, and will not be discussed here. Visual loss, periocular pain and dyschromatopsia make up the three most common symptoms, however not all patients present with all three complaints. The visual loss is commonly unilateral, varies in severity from mild to severe, with total loss of light perception, and progresses for an average of seven to ten days. Ninety percent of patients with optic neuritis have periocular pain, which may occur before the onset of vision loss.^1^ However, patients may not initially complain of pain. It is helpful to specifically ask if there is pain with eye movements, which is thought to occur as the inflamed optic nerve is stretched with globe motion. Color and contrast sensitivity are often decreased out of proportion to loss of visual acuity.^6^ Visual field loss, especially a central scotoma, is typical in optic neuritis.^7^

A relative afferent pupillary defect is present in the affected eye unless there is symmetric involvement or history of optic neuropathy in the fellow eye.^6^ Sixty-five percent of patients have normal optic nerve appearance on funduscopic examination at presentation; these patients are considered to have retrobulbar optic neuritis.^1^ The optic disc may be swollen, but prominent swelling of the nerve head, the presence of exudates, or marked hemorrhage may suggest other diagnoses.^4^ Optic disc pallor typically develops four to six weeks after the onset of inflammation.

Several other findings have been described in optic neuritis, though they are not sensitive indicators of the condition. These include Uhthoff’s phenomenon which describes worsening of vision with increased body temperature, and the Pulfrich phenomenon, in which a simple pendulum swinging in one plane appears to trace an ellipse due to asymmetrical optic nerve conduction velocity.^1^

### Distinguishing Features from Other Optic Neuropathies

Since the diagnosis of optic neuritis is clinical, it is useful for physicians to understand other processes which cause similar findings. The differential diagnosis for optic neuritis (defined as optic nerve inflammation) is very broad, including other auto-immune neuropathies (e.g., systemic lupus erythematosus), rheumatologic conditions, inflammatory conditions (e.g., sarcoidosis), compressive neuropathies (e.g., meningioma), infections (e.g., sinusitis), and toxic (e.g., ethambutol) or nutritional neuropathies.

**Figure 1:**
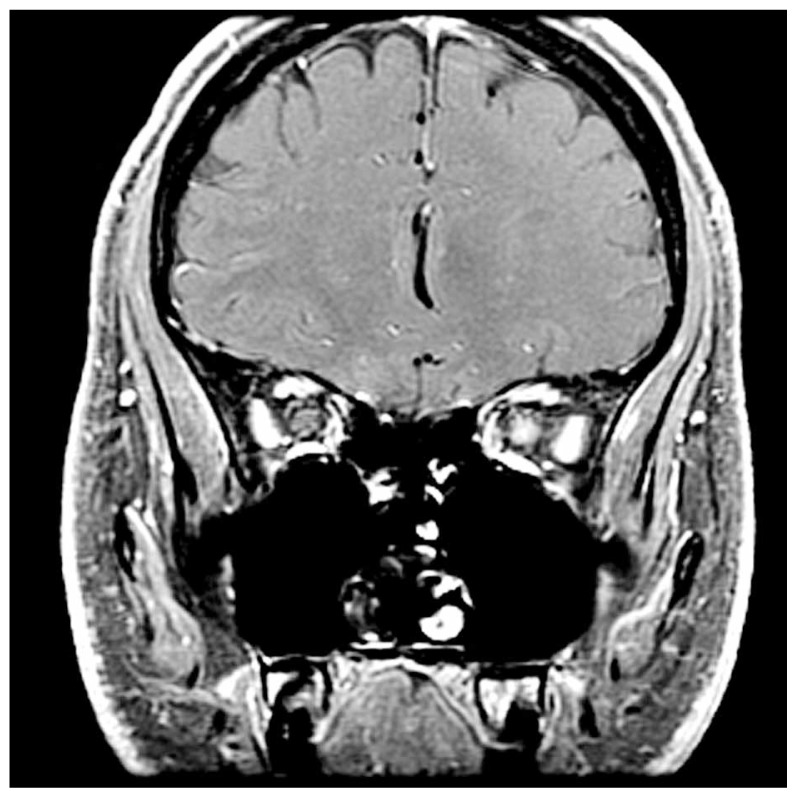
Coronal T1 contrast-enhanced fat suppressed magnetic resonance imaging showing enhancement of the left optic nerve (arrow) which is typical of optic neuritis.

Two conditions which are thought to be non-inflammatory and most closely resemble the clinical profile of optic neuritis are anterior ischemic optic neuropathy (AION) and Leber hereditary optic neuropathy (LHON). While sudden unilateral visual loss with optic disc swelling is present in AION, the patients are typically over 50 years of age (without a female preponderance) and less commonly have pain with eye movement.^21^ By definition, the optic disc must be swollen acutely in AION, and the swelling is often segmental with hemorrhage.^7^ LHON, like optic neuritis, may cause visual field loss with a central scotoma, impaired visual acuity and poor color vision. However, it occurs most commonly in young men (eighty-five percent of the time), is not typically accompanied by pain and usually progresses over weeks to months to involve both eyes sequentially.^4^ Because of the mitochondrial inheritance pattern, there is often a family history of subacute visual loss in maternally related men. Fat suppressed orbital magnetic resonance imaging (MRI) typically shows enhancement of the optic nerve in optic neuritis (Fig. 1), but not so in AION or LHON.

### Brain MRI

**Figure 2:**
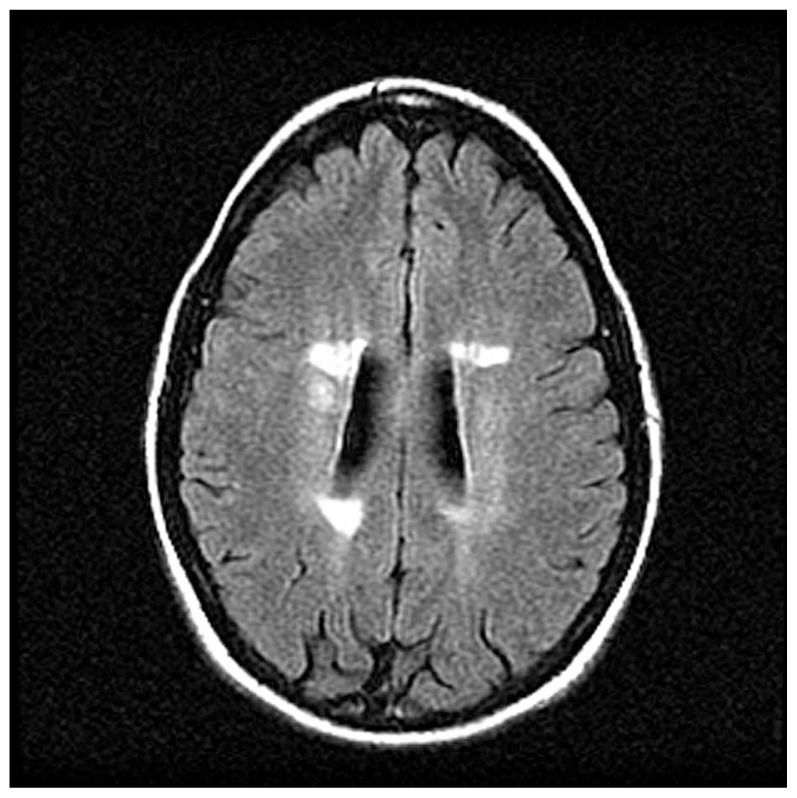
Axial magnetic resonance imaging fluid-attenuated inversion recovery (FLAIR) sequence demonstrates periventricular lesions typical of demyelination from multiple sclerosis.

**Figure 3:**
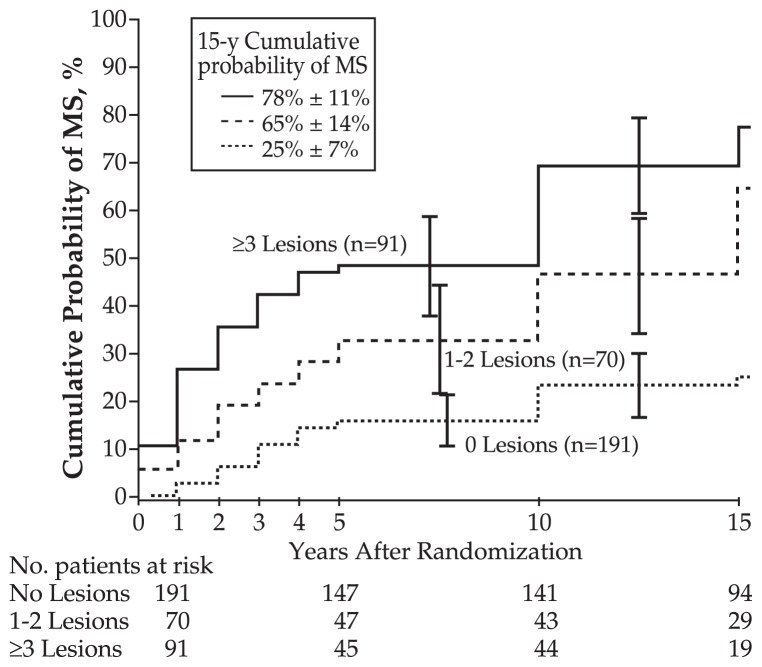
Life-table analysis of multiple sclerosis (MS) according to the number of lesions on baseline brain magnetic resonance imaging (MRI). Life-table intervals are defined by annual examinations during the first 5 years of the study and periods after the 5- and through the 10-year examinations and after the 10- and through the 15-year examinations. The table under the horizontal axis represents the number of patients during follow-up who had not developed MS at the end of the previous interval. Patients with 1 or 2 lesions on MRI were combined into a single group because the rates of MS were similar. From “Multiple sclerosis risk after optic neuritis: final optic neuritis treatment trial follow-up. Arch Neurol 2008;65:727-732.”^8^

Because of the known association between optic neuritis and multiple sclerosis, patients without a known history of multiple sclerosis are typically offered a brain MRI. The Optic Neuritis Treatment Trial is one study that has helped stratify the risk of developing multiple sclerosis on the basis of abnormal findings on brain MRI (Fig. 2). A patient with a first episode of optic neuritis but normal brain MRI has a 25% chance of developing multiple sclerosis at fifteen years, as opposed to a 72% risk for patients with one or more lesions (Fig. 3).^8^ Other studies have shown that more detailed early MRI findings such as baseline lesion number, location, and activity; three-month lesion activity measures; and brain atrophy, magnetization transfer ratio, and spectroscopy measures may improve the prediction of development of multiple sclerosis. New T2 lesions on an early follow-up scan have been suggested as the strongest independent predictor of multiple sclerosis.^9^ Although additional predictions can be made based on gender, race, age, family history, and location of the swelling along the optic nerve, the number of lesions on initial brain MRI is most strongly related to the risk of developing multiple sclerosis.^1^,^8^

### Other Tests

**Figure 4:**
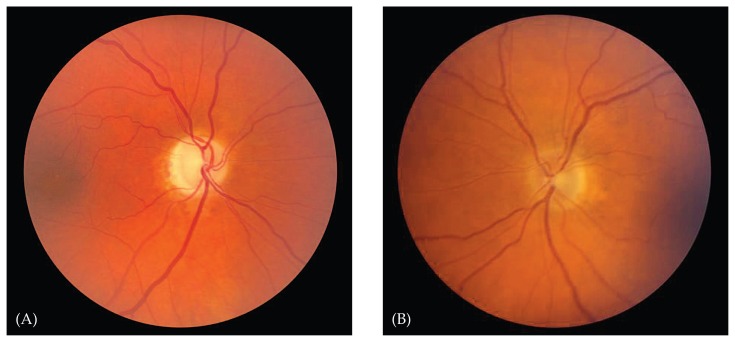
A 45 year-old man with prior optic neuritis in the right eye. The right optic disc **(A)** is pale compared to the normal left optic disc **(B)**.

**Figure 5:**
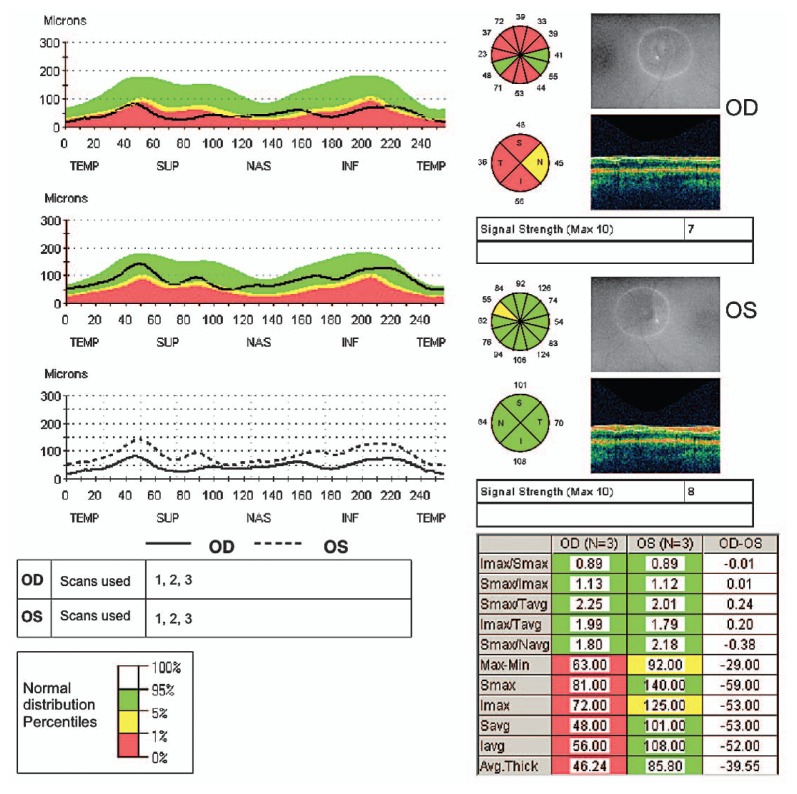
Optical coherence tomography shows thinning of the retinal nerve fiber layer of the right eye in the same patient as in Fig. 4.

Other diagnostic tests do not have such clear support. Lumbar puncture for cerebrospinal fluid (CSF) analysis, for example, is recommended by some to look for the oligoclonal banding typical of multiple sclerosis.^22^ Others have noted that patients with oligoclonal CSF bands are typically the same patients with an abnormal MRI, and that lumbar puncture should be reserved for patients in whom the diagnosis of optic neuritis is in question.^10^ Visual evoked potentials (VEPs), including multifocal VEPs, are recommended by some authors as a sensitive and specific tool for detecting demyelinating optic neuritis,^11^ while others report the test not to be helpful in differentiating the cause of optic neuropathy in its acute phase, reserving the test for subclinical cases in which poor color vision or a pale optic disc are noted.^1^ Other tests, such as optical coherence tomography, may be useful in documenting thinning of the retinal nerve fiber layer after an episode of optic neuritis (Figures 4, 5), and its clinical utility is expanding.^12^

## MANAGEMENT

### Optic Neuritis Treatment Trial

Once a diagnosis of optic neuritis has been made and the risk of future development of multiple sclerosis has been estimated on the basis of a brain MRI, patients will likely ask about treatment options. The largest study to address this question is the Optic Neuritis Treatment Trial, a multicenter randomized clinical trial with fifteen years of follow up. Patients were assigned to three groups: one received oral prednisone, another received intravenous methylprednisolone followed by an oral prednisone taper, and the last received oral placebo.

The trial showed that intravenous corticosteroids followed by an oral taper accelerated visual recovery, but did not improve the long-term visual outcome when compared to placebo. Patients who were treated with oral corticosteroids alone did not have improved visual outcomes and were actually shown to have an increased rate of recurrent optic neuritis in the same or fellow eye.^3^ Thus viable options for treatment of an initial bout of optic neuritis include either treatment with intravenous corticosteroids followed by an oral steroid taper, or no treatment at all. Oral corticosteroids alone at doses of 1 mg/kg do not have a beneficial role. Interestingly, the treatment of other clinical presentations and exacerbations of multiple sclerosis is based on this data even if the exacerbation does not involve vision. This is likely not only because optic neuritis is such a common finding in patients with multiple sclerosis, but also because it is the most consistent and thoroughly defined event associated with the disease.

### Long-Term Management

The presentation of demyelinating optic neuritis often invites referral to a neurologist familiar with the diagnosis and treatment options. Data from the Optic Neuritis Treatment Trial have shown that patients who present with acute demyelinating optic neuritis have a favorable long-term visual prognosis, even if they develop multiple sclerosis.^13^

Long-term treatments for optic neuritis include the interferons, often used in patients with multiple sclerosis. The neurologic literature has shown that patients treated with immunomodulating agents have improved clinical outcomes and neuroimaging abnormalities compared to those who are not treated.^14^,^15^ Furthermore, use of interferons has been associated with a lower risk of subsequent multiple sclerosis in patients with an acute neurologic syndrome, including optic neuritis.^16^

### Unanswered Questions

Several unanswered questions remain regarding the presentation, treatment, and implications of optic neuritis. Studies have shown that the risk of developing clinically definite multiple sclerosis after presenting with a clinically isolated syndrome such as optic neuritis is increased in patients with an abnormal brain MRI, and evidence supports treatment of these patients with immunomodulating agents.^14^-^16^ Patients with a normal brain MRI still have a 15-22% risk of developing multiple sclerosis, but as the management of a patient with a first episode of optic neuritis and a normal brain MRI has not been studied extensively, it remains controversial.^17^-^19^ Furthermore, while the treatment of acute demyelinating optic neuritis with intravenous corticosteroids followed by an oral corticosteroid taper is supported by evidence from the Optic Neuritis Treatment Trial,^3^ the role of high dose oral corticosteroids in optic neuritis has not been systematically studied. The value of corticosteroids in patients with recurrent optic neuritis is also undetermined at this time.

Large clinical trials with long-term follow-up have provided invaluable insights on the clinical manifestations, management, prognosis, and implications of an initial bout of acute demyelinating optic neuritis, but the remaining uncertainties highlight the need for continued research.
